# Refractive outcomes of small lenticule extraction (SMILE) Pro® with a 2 MHz femtosecond laser

**DOI:** 10.1007/s10792-024-02915-2

**Published:** 2024-02-10

**Authors:** Amr Saad, Karsten Klabe, Mücella Kirca, Florian A. T. Kretz, Gerd Auffarth, Detlev R. H. Breyer

**Affiliations:** 1 Breyer, Kaymak & Klabe Eye Surgery, Martin-Luther-Platz 22, 40212 Duesseldorf, Germany; 2 Institution for International Innovative Ophthalmic Surgery, Duesseldorf, Germany; 3 Precise Vision, Kretz & Colleagues, Rheine, Germany; 4 Department of Ophthalmology, University Hospital Heidelberg, Heidelberg, Germany

**Keywords:** Small incision lenticule extraction, Smile pro, Carl Zeiss, VisuMax 800, Safety, Efficacy, Predictability, Accuracy

## Abstract

**Purpose:**

To evaluate the initial visual outcomes of Small Incision Lenticule Extraction (SMILE) Pro® using a 2 MHz femtosecond laser (VisuMax 800, Carl Zeiss Meditec) and to assess the efficacy, safety, predictability, accuracy, and complication rate.

**Methods:**

This retrospective analysis included eyes which underwent the SMILE Pro® procedure using VisuMax 800 femtosecond laser to correct myopia. All surgeries were performed by one surgeon (DB). Follow-up was conducted 3 months postoperatively to evaluate visual outcomes after neuroadaptation, corrected visual acuity (CDVA) and intra- and postoperative complications.

**Results:**

One hundred and fifty-two eyes of 82 patients (mean age 31 ± 6 years) results at 3 months are presented. The mean spherical equivalent (SE) was − 4.44 ± 1.86 D preoperatively while -0.24 ± 0.32 D postoperatively. 99% of eyes achieved SE within ± 1.0 D of attempted correction and 91% were within ± 0.5 D. Efficacy index was 0.93 while the safety index was 1. No complications occurred intra- or postoperatively. No eyes lost more than 1 line of their preoperative CDVA. All highly myopic eyes (− 6.25 to − 10.00 D; *n* = 18) achieved 20/20 at 3 months postoperatively and were within 0.5 D from the attempted SE and no eyes lost more than 1 line of CDVA.

**Conclusion:**

The SMILE Pro® is a safe, efficient, and predictable procedure for the treatment of myopia and myopic astigmatism, with comparable results of conventional SMILE surgery. High myopic eyes achieve better results than low and moderate myopia. No complications were recorded in our patients.

## Introduction

Over the past decade, refractive lenticule extraction (ReLEx) has evolved to become one of the latest developments in the area of minimally invasive keratorefractive surgery [1]. Small Incision Lenticule Extraction (SMILE) was first performed in 2007 by Sekundo et al. [2] and has progressed as a flapless procedure surgery in which an intrastromal lenticule is created by a femtosecond laser and then manually extracted through a small corneal incision that is created peripherally [2, 3]. This method eliminates flap-related complications and appears to be safe and effective, independent of the environmental conditions [4, 5].

With a high level of biomechanical stability of the cornea in the postoperative period, it has reduced the risk of postoperative ectasia compared to the flap-based LASIK surgery [2, 6]. Moreover, it ensures stability of the ocular surface and a more rapid regeneration of corneal nerves, resulting in a lower incidence of iatrogenic dry eye symptoms and postoperative trauma complications [4]. Additionally, it is an all-in-one corneal procedure that uses only one femtosecond laser, reducing surgery effort compared to LASIK, which also relies upon an excimer laser [2, 7].

Good outcomes and promising long-term clinical results have been presented in several peer reviewed studies [4, 8, 9]. A recently published meta-analysis showed that SMILE surgery appears to be on par with Femtosecond LASIK for myopia correction in terms of safety, efficacy, and predictability [10].

Notwithstanding, SMILE is not without limitations: cases of suction loss have been recorded at variable rates [11–13], and SMILE is still unable to correct higher order aberrations [14].

In late 2021, the latest laser platform of this sort was introduced, the VisuMax 800 (Carl Zeiss Meditec, Jena, Germany), which is claimed to offer new advances in surgical performance by reducing treatment time and risk of suction loss. While the previous version, VisuMax 500, runs at 500 kHz, the new version runs at 2 MHz, which results in less time (30 versus 10 s) needed for lenticule creation and consequently, less time required for the patient to remain still, which leads to better patient experience [15].

In this study, we report the clinical results of the first treated eyes with VisuMax 800 in our eye laser center, evaluating efficacy, safety, predictability, and accuracy.

## Methods

### Study design

This retrospective analysis (performed in the course of our quality management introducing new surgical procedures) included 152 eyes of 82 patients who underwent SMILE Pro^®^ surgery for correction of myopia and myopic astigmatism from January 2022 to July 2022 at Breyer, Kaymak & Klabe Eye Surgery and Premium Eyes clinic in Duesseldorf, Germany. Some patients only received treatment to one eye for intended monovision. This investigation is in accordance with the principles of the Declaration of Helsinki, and since the surgeries were done routinely as standard practice using a CE Marked device, ethical approval was not required. After detailed patient education, all participants provided written informed consent for undergoing laser surgery and inclusion of their data for research purposes.

### Patients

Inclusion criteria included spherical myopia from − 1 D to − 10.00 D and myopic astigmatism up to 5.00 D. Patients had to have a 2-week contact lens break prior to preoperative measurements and surgery. Corrected distance visual acuity (CDVA) was at least 20/25 (LogMAR: 0.097) in each eye and refraction had to be stable for at least 2 years. All patients were older than 18 years and able to sign an informed consent. Exclusion criteria included any ocular surgery or trauma as well as systemic diseases that might affect the eye. Moreover, a history of ocular inflammation, retinal detachment, any form of corneal disease including corneal ectasia, an endothelial cell count less than 1900/mm^2^, and manifest dry eye led to exclusion from this analysis. Eyes with more than 40% of percentage tissue altered (PTA) [defined as: PTA = (lenticule thickness + cap thickness)/central corneal thickness)] were also excluded as it is suggestive of an increased risk of ectasia [16]. Postoperative residual stromal bed had to be greater than 250 μm. In addition, the instructions of the manufacturer regarding contraindications were taken into consideration [17].

Prior to surgery a detailed ocular examination was performed on the eyes of all subjects, including slit-lamp examination, indirect ophthalmoscopy, tonometry, and specular microscopy. Additionally, tomographic measurements using a scheimpflug-based system (Pentacam HR, Optikgeräte GmbH, Wetzlar, Germany), and a wavefront analysis (KR-1W, Topcon Corp., Tokyo, Japan) were performed. Objective and manifest refractions were assessed, as well as uncorrected distance visual acuity (UDVA) and corrected distance visual acuity (CDVA).

### Surgery

All SMILE Pro procedures were performed by the same experienced surgeon (DB) using a VisuMax 800 femtosecond laser system (Carl Zeiss Meditec AG) with a repetition rate of 2 MHz following a standard surgical technique [15]. After applying topical anesthetic eye drops (0.4 mg Conjuncain, Bausch & Lomb Inc., USA) and positioning the head correctly, the surgeon set the correct pupil centering and then docked the curved contact glass to the cornea. A size “S” suction contact glass interface was used for all treated eyes. The patients were asked to fixate on a flashing light during docking to avoid decentration. During the docking process, the vector difference between corneal vertex and the current position of the eye was presented by the CentraLign assistant function using data from corneal topography (IOL Master 700). The surgeon can align the treatment center with the corneal vortex using joystick. Subsequently, suction was initiated and an intrastromal lenticule was created automatically by the femtosecond laser. A thin hooked instrument (Breyer-Pfäffl Spatula, Geuder Inc, Germany) was used to separate the refractive lenticle from the surrounding stroma which was then extracted through the peripheral corneal incision using a microforceps. Both eyes were treated consecutively in the same session, if bilateral treatment was the goal. Immediately after surgery, the surgeon examined all patients with a slit lamp. The surgical parameters of the VisuMax 800 were a cap thickness between 120 and 150 μm and a diameter of 7.6 mm. The optical zone was between 6.5 and 7.0 mm with a transition zone of 0.1 mm. All side-cut angles were 90° at a position of 180°, while the incision width was 2.92 mm. Suction time was 9 or 10 s and the pulse duration was between 220 and 580 fs. Each surgery was recorded in case of possible complications to be documented.

All patients were prescribed Ofloxacin (Ratiopharm Ltd., Germany) and Efflumidex® (Allergan Pharmaceuticals, Ireland) eye drops five times a day for 2 days before and for 1 week after surgery. Artificial tears, Remogen® eye drops (TRB Chemedica Inc, Germany), were also prescribed to be used for 2 weeks before the treatment and then adjusted to each individual patient’s symptoms postoperatively. During the 3-month follow-up, CDVA and UDVA was measured in all patients, eyes were examined for any postoperative complications and patient complaints were documented.

### Outcome measurement

The main outcome of this study is to assess the visual, refractive, and safety outcomes of the VisuMax 800 3 months postoperatively to allow for neuroadaptation [18]. Clinical results were presented in accordance with the Standard Reporting in Refractive Surgery described by Reinstein et al. [19]. Microsoft Excel templates developed by the London Vision Clinic were used to create the charts. The efficacy index (EI) describes the ratio between postoperative UDVA and preoperative CDVA (converted in decimal notation), whereas the safety index (SI) is defined as the ratio between postoperative CDVA and preoperative CDVA (converted in decimal notation). The predictability graphs demonstrate an evaluation of how close we could achieve an intended target spherical equivalent (SE) whereas the accuracy is evaluated as the proportion of eyes achieving a postoperative SE within ± 0.50 or ± 1.0 D of intended target. The preoperative and postoperative data were collected from the electronic medical records and imported into an Excel spreadsheet (Microsoft Corporation, Redmont, USA). For all tests, a p value less than 0.05 was considered statistically significant.

## Results

Our investigated cohort consisted of 152 eyes from 82 patients; 35 men (43%) and 47 women (57%), and the mean age was 31 ± 6 years. All eyes underwent SMILE surgery in the same session but 12 patients who underwent SMILE Pro in one eye only. The mean spherical equivalent (SE) was − 4.44 D ± 1.86 D preoperatively while − 0.24 D ± 0.32 D postoperatively. The patients were divided into three groups based on their preoperative spherical equivalent. The low myopia group (− 1.00 to − 3.00 D) consisted of 46 eyes (31%), the moderate myopia group (− 3.25 to − 6.00 D) consisted of 84 eyes (57%), and the high myopia group (− 6.25 to − 10.00 D) included 18 eyes (12%). Four eyes with a SE lower than 1 D were not in included in the subgroup analysis. All eyes were targeted for emmetropia and the follow-up was at 3 months after surgery.

### Efficacy

**Fig. 1:**
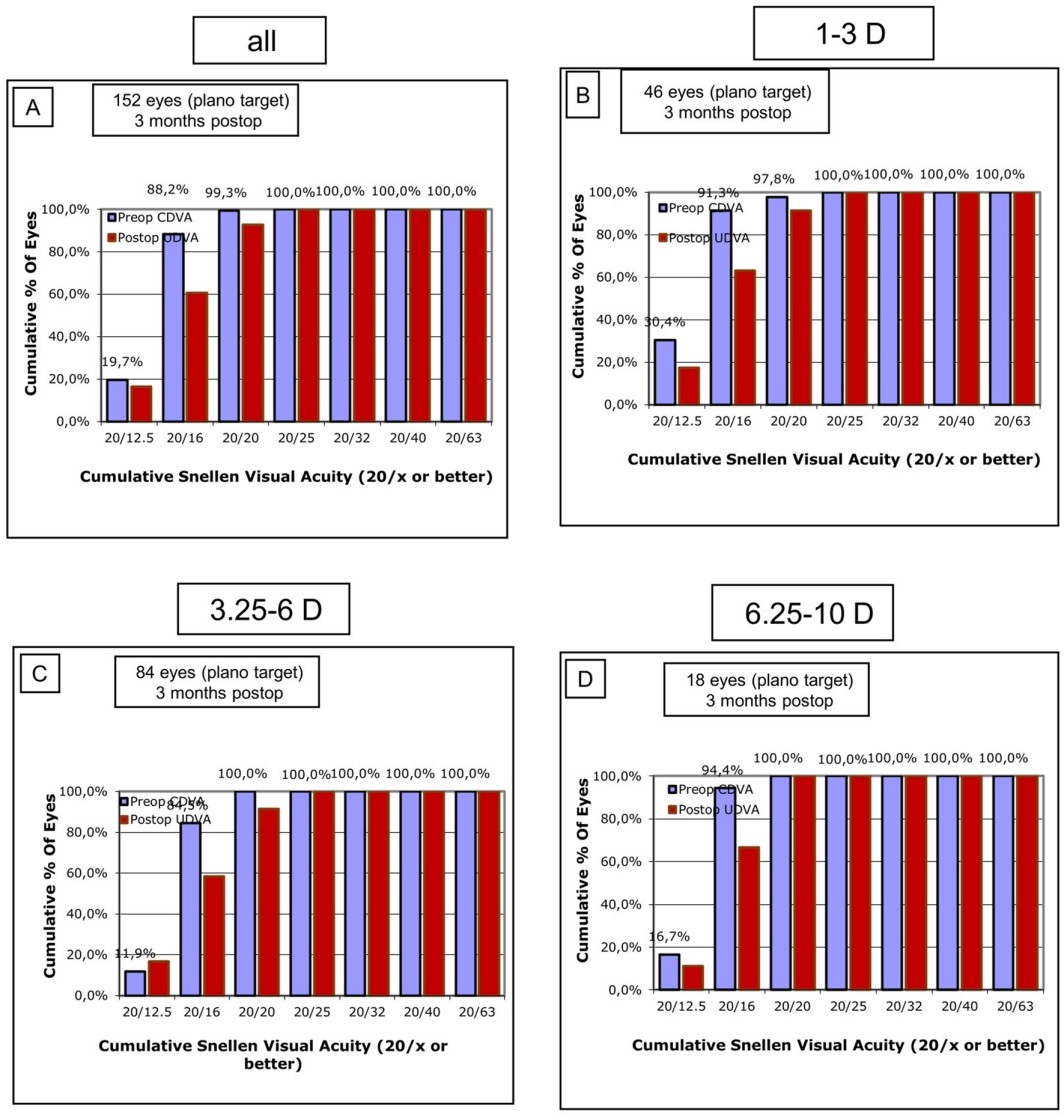
Efficacy Graphs reporting preoperative CDVA in relation to postoperative UDVA for the overall group (**a**) and the three subgroups (**b**–**d**): postop: postoperatively, CDVA: corrected distance visual acuity, UDVA: uncorrected distance visual acuity, D: diopters

The efficacy of the procedure was determined based on the postoperative uncorrected distance visual acuity (UDVA) in relation to the preoperative corrected distance visual acuity (CDVA). The efficacy index (EI) was calculated as the ratio between the mean postoperative UDVA and the mean preoperative CDVA of all subjects. The mean preoperative CDVA was − 0.09 ± 0.05 logMAR, the mean postoperative UDVA was − 0.06 ± 0.08 logMAR, and the efficacy index was 0.93. Furthermore, in the overall group, 92.8% of eyes achieved emmetropia (20/20 Snellen lines of UDVA) compared to 99.3% of eyes with 20/20 in preoperative CDVA. Figure 1 illustrates the cumulative Snellen postoperative UDVA versus preoperative CDVA for the entire cohort and the subgroups. The graphs indicate that the procedure was particularly effective in the high myopia group (− 6.25 to − 10.00 D), where all eyes achieved a postoperative UDVA of 20/20 Snellen lines (Fig. 1d).

### Safety

**Fig. 2:**
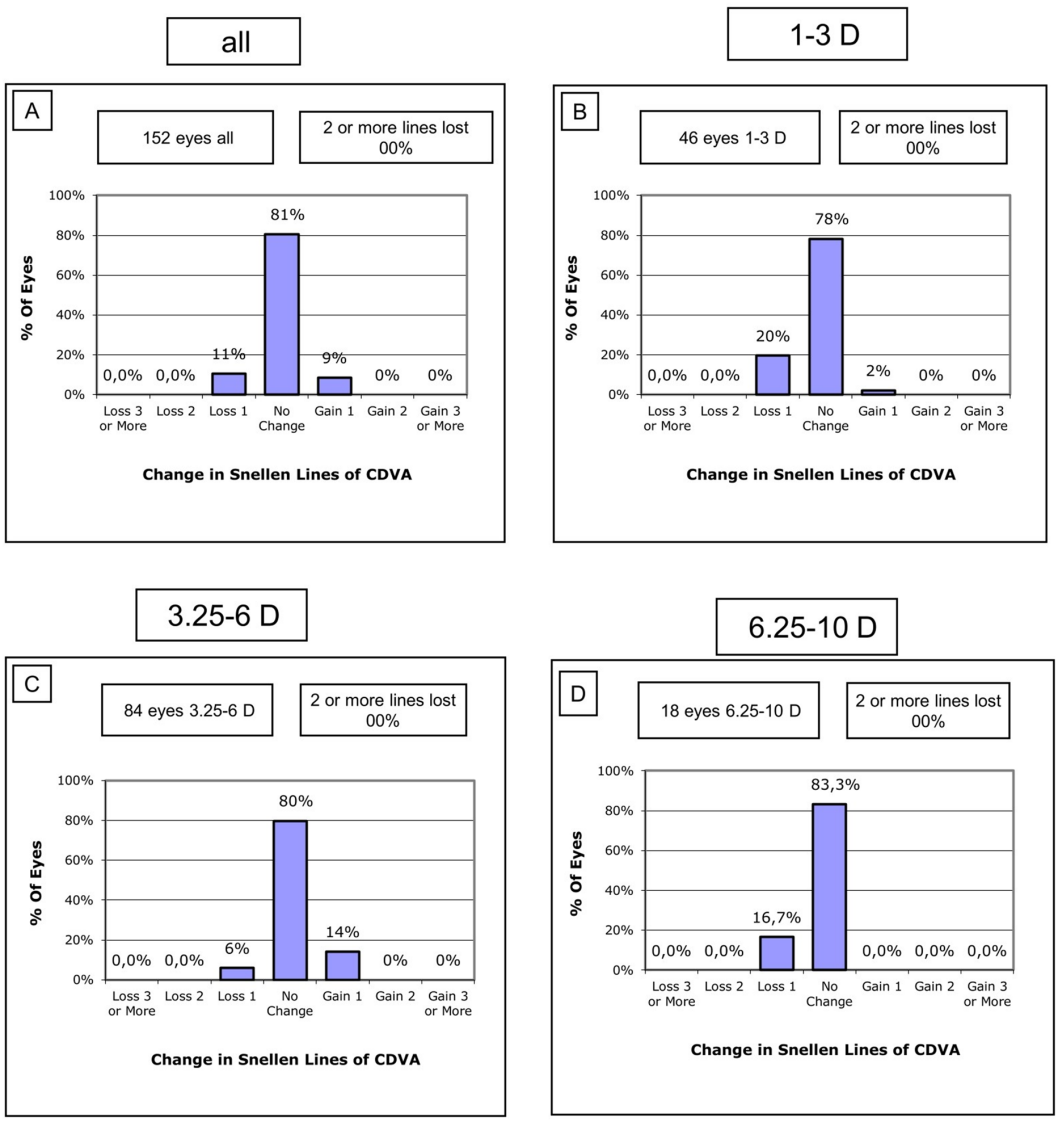
Safety Graphs reporting the change in Snellen Lines of CDVA for the overall group (**a**) and the three subgroups (**b–d**): postop: postoperatively, CDVA: corrected distance visual acuity, D: diopters

The safety was assessed based on the postoperative change in CDVA. A loss of two or more Snellen lines is generally considered to have a significant impact and to be noticeable for the patient. The mean preoperative CDVA was − 0.09 ± 0.05 logMAR while the mean CDVA 3 months postoperatively was − 0.09 ± 0.07 logMAR, and the safety index was 1. Figure 2 presents the changes in Snellen lines. The highest proportion of eyes gaining one Snellen line was observed in the moderate myopic eyes (− 3.25 to − 6.00 D) with 12 eyes (14%, Fig. 2c), while the highest proportion with a loss of one Snellen line was seen in the low myopia group (− 1.00 to − 3.00 D) with 9 eyes (20%, Fig. 2b). However, most of the eyes (81%) showed no change in CDVA (Fig. 2a). No patients experienced a loss of two or more lines of CDVA and no complications or abrasion, abandoned procedure, or inflammation occurred during or after surgery, indicating a high level of safety for this procedure. Notably, no loss of suction was observed during all surgeries.

### Predictability

**Fig. 3:**
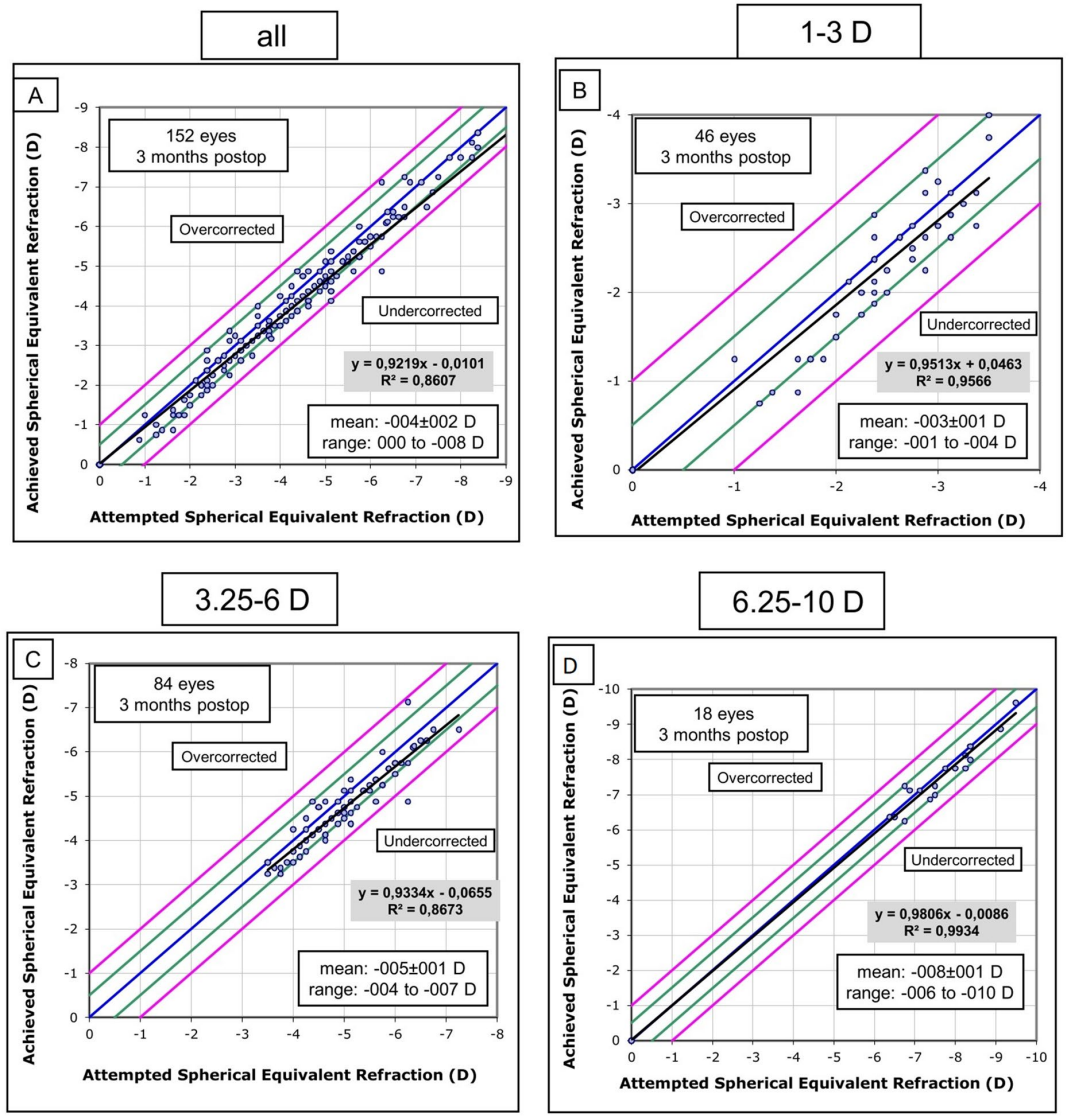
Predictability Graphs reporting attempted vs achieved SE refraction for the overall group (**a**) and the three subgroups (**b**–**d**): postop: postoperatively, SE: spherical equivalent, *R*^2^: Coefficient of Determination, D: diopters

To assess the predictability of refractive outcomes, we analyzed the attempted and achieved spherical equivalent (SE) refraction. Figure 3 displays a scatterplot of attempted versus achieved correction in terms of SE. Overall, our data showed a slight under-correction with a coefficient of determination (*R*^2^ = 0.86) for the entire group (Fig. 3a). Notably, we found a slight under-correction in the moderate myopia group (− 3.25 to − 6.00 D; 55% of the cohort) which was reflected in a lower regression (*R*^2^ = 0.87, Fig. 3c) compared to the other two subgroups, whereas *R*^2^ was 0.96 and 0.99 in the low and high myopia groups, respectively. (Fig. 3b and d).

### Accuracy

**Fig. 4:**
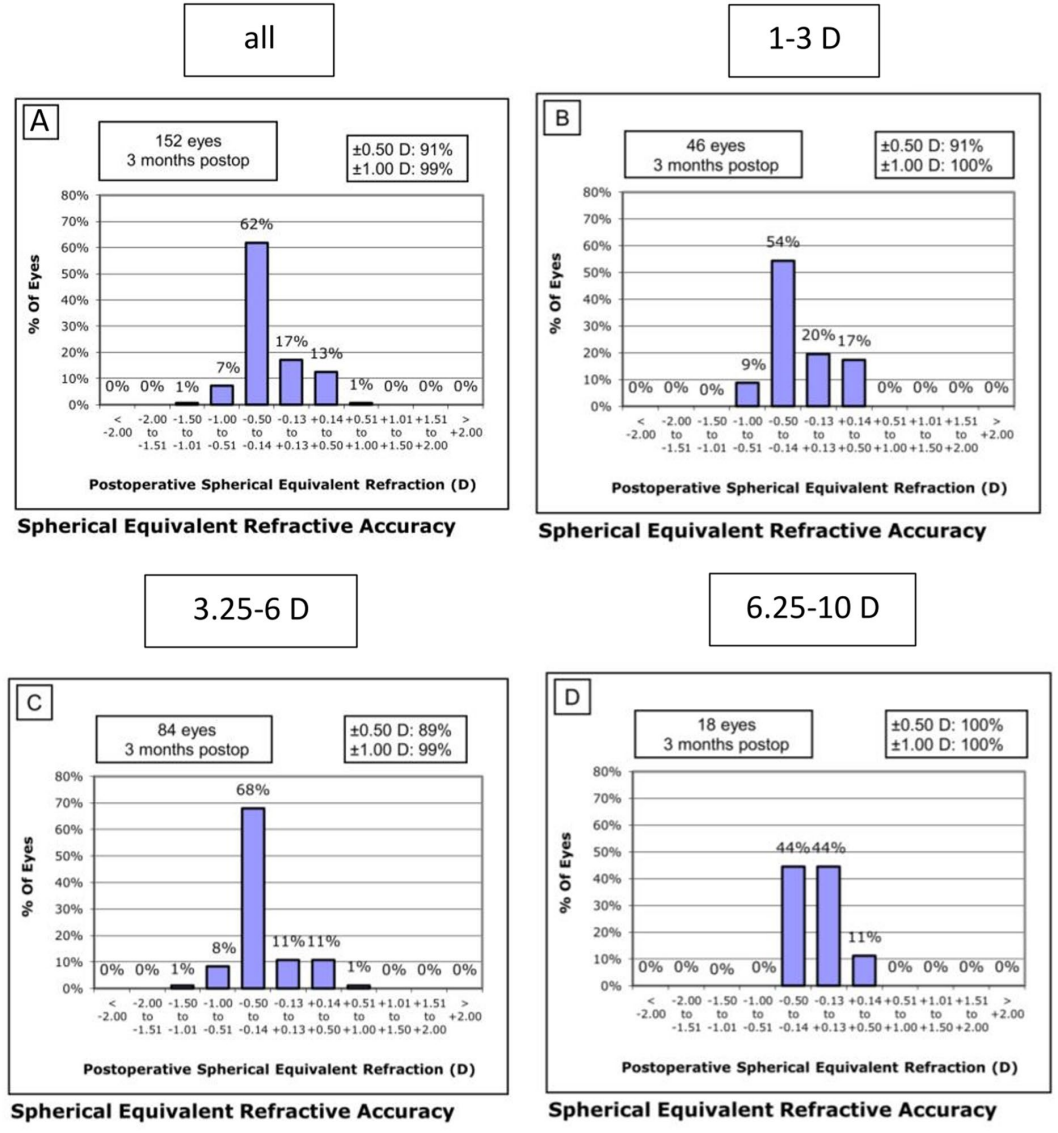
Accuracy Graphs reporting the proportion of eyes that achieved a certain level of postoperative SE within specific ranges for the overall group (**a**) and the three subgroups (**b**-**d**): postop: postoperatively, SE: spherical equivalent, D: diopters

We assessed the accuracy of the refractive outcome by calculating the proportion of eyes achieving a SE within specific ranges of the target refraction. The results of this analysis are presented in Fig. 4. Our findings show that 91% of eyes achieved a SE within ± 0.50 D, while 99% achieved a SE within ± 1.0 D of the target refraction after a postoperative follow-up period of 3 months, indicating a very high accuracy of the procedure (Fig. 4a).

### Refractive astigmatism

**Fig. 5:**
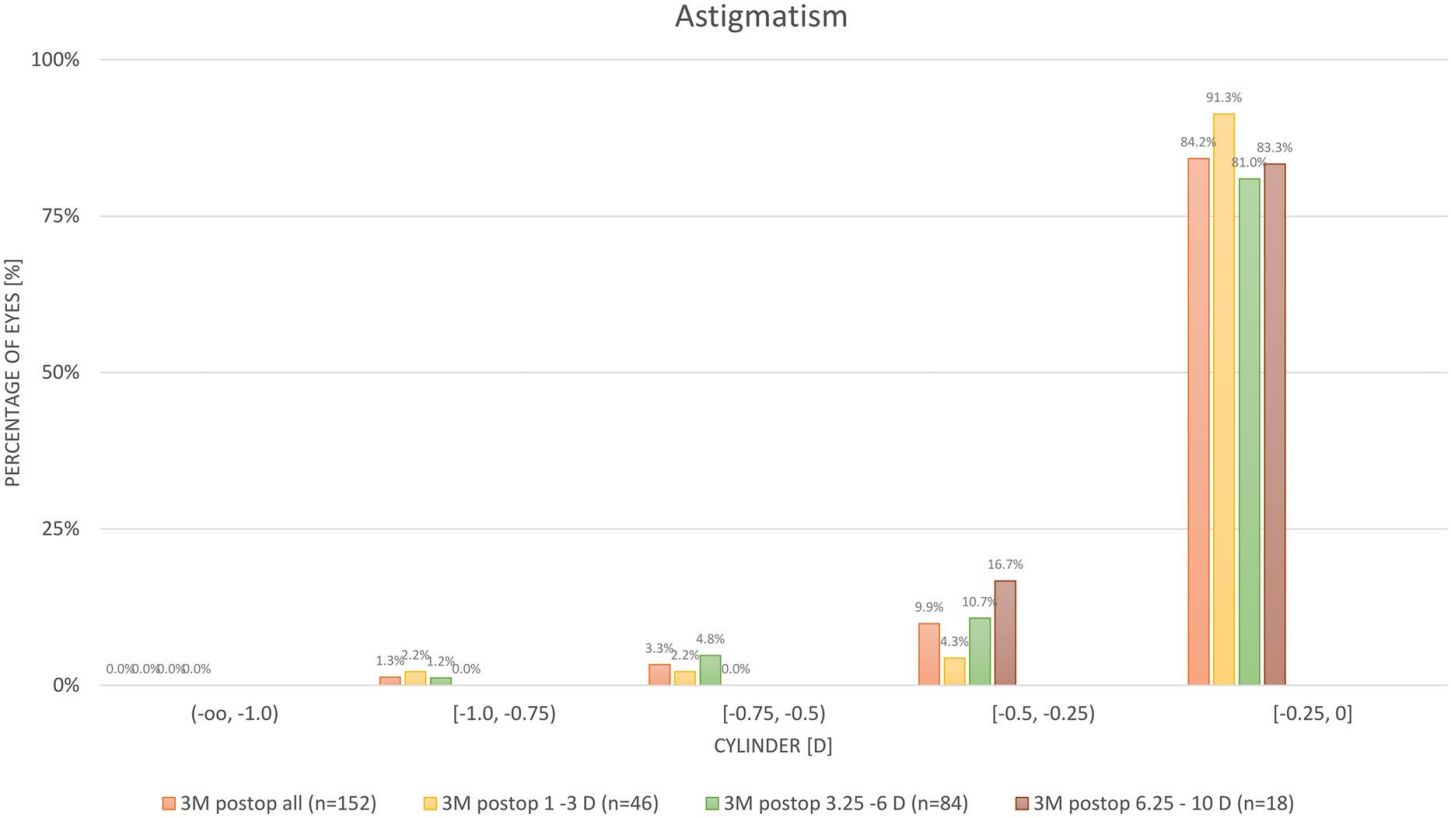
Residual Astigmatism in the whole cohort as well as the subgroups

Figure 5 shows residual postoperative astigmatism for the whole cohort and the three subgroups. Overall, 95% of eyes had residual astigmatism within 0.5D. Ninety-one percent of the low astigmatism group had residual astigmatism within 0.25D.

## Discussion

The increasing demand for flap-free refractive surgery, driven by the growing degree of patient awareness regarding the potential flap-associated risks, along with the desire to maintain an active lifestyle, is subsequently leading to improvements in the femtosecond laser systems. Previous studies have demonstrated that the ReLEx® SMILE is a flapless, minimally invasive alternative to traditional flap-based procedures with high levels of efficacy, predictability, and safety [3, 20–22]. Our study builds upon previous findings, confirming the safety of SMILE surgery showing a lower incidence of perioperative complications compared to flap-based methods and resulting in a reduced risk of postoperative dry eye combined with a greater biomechanical strength [4, 21].

The recently introduced VisuMax 800 laser platform has shown highly promising outcomes. Our study revealed visual outcomes that were comparable or superior to the previous laser generation, VisuMax 500 [2, 3, 23, 24].

Our results are comparable with the results of a recent study by Reinstein et al. [15], who performed SMILE Pro using VisuMax 800 on 128 eyes of 66 patients. Three-month postoperative UDVA was 20/20 in 91% of eyes in their study and 93% in our study. Postoperative spherical equivalent in their study was within ± 0.50 D in 86% of eyes and within ± 1 D in 100% of eyes, while in our study it was within ± 0.50 D and ± 1 D in 91% and 99%, respectively. There was a gain of CDVA in 15 and 9% in their study and our study, respectively, while 68 and 81%, respectively, stayed the same, and 8 and 11%, respectively, lost one line with no eyes losing more than one line in both studies. Additionally, 84 and 95%, respectively, had residual astigmatism within 0.5 D.

SMILE may be considered by some authors to be a better option for high myopia than LASIK given that it is flap-free and it has been shown to produce good visual quality in high myopia [25, 26]. Previous short-term studies (up to 6 months) using VisuMax 500 found that between 37 and 80% of eyes achieved UDVA of 20/20 and less than 20% lost more than one line of CDVA [21, 27–29]. The results of the current study compare favorably to these studies. In the current study, 100% of eyes (*n* = 18) in the high myopia group achieved 20/20 3 months post operatively with remarkable predictability *(R*^2^ = 0.99), and accuracy (100% of eyes were within 0.5 D from the attempted SE and 44% were within 0.13 from the attempted SE). However, long-term studies are needed to assess the possible regression commonly noticed in high myopia after laser refractive surgeries [29].

Several studies have investigated intraoperative suction loss that can result in procedure cancellation and the need for retreatment [30–33]. Ang et al. [34] reported that suction loss occurred in 2/70 (3%) in the SMILE group using VisuMax 500, while in the current study, none of the eyes experienced suction loss. A previous study by Reinstein et al. estimated that 65% of suction loss cases occurs during creation of the lenticule interface after 10 s [13], and since suction time is reduced with VisuMax 800 down to 10 s, Reinstein et al. anticipated that the incidence of suction loss should decrease by 65% [15].

Shorter treatment times with the new VisuMax 800 may reduce patients’ anxiety and eye movements during surgery, which have been linked to intraoperative complications [4, 11, 13, 33, 35, 36]. Furthermore, the integrated cyclotorsion tracking system (OcuLign) in the VisuMax 800 may eliminate the need for manual readjustment and reduce problems with decentration in the near future [37, 38]. On the other hand, a slight under-correction was observed in the moderate myopia group and this is consistent with the fact that SMILE may be slightly less accurate than LASIK in treating low to moderate refractive errors [39].

Our study had some limitations due to its retrospective design and a short follow-up period of 3 months. Long-term studies are needed to investigate any regression and to determine whether using the VisuMax 800 can offer significant benefits in addressing the challenges and complications associated with the conventional SMILE surgery. This study aimed to analyze the visual and refractive outcomes of this new laser platform and suggests comparable results with the previous VisuMax 500 laser platform.

Small lenticule extraction Pro, using VisuMax 800, was shown to have comparable efficacy, safety, and accuracy to SMILE using VisuMax 500. The high myopia group achieved particularly good results using VisuMax 800.The shorter treatment time and a reduced rate of suction loss and complications also led to improved patient experience.
